# Prevalence Characteristics of Coal Workers’ Pneumoconiosis (CWP) in a State-Owned Mine in Eastern China

**DOI:** 10.3390/ijerph120707856

**Published:** 2015-07-10

**Authors:** Lei Han, Ruhui Han, Xiaoming Ji, Ting Wang, Jingjin Yang, Jiali Yuan, Qiuyun Wu, Baoli Zhu, Hengdong Zhang, Bangmei Ding, Chunhui Ni

**Affiliations:** 1 Department of Occupational Medicine and Environmental Health, School of Public Health, Nanjing Medical University, Nanjing 211166, China; E-Mails: hanlei@jscdc.cn (L.H.); hanruhui007@163.com (R.H.); jxmnjmu@163.com (X.J.); wangti08@163.com (T.W.); njmujj@126.com (J.Y.); yjlgogo@sohu.com (J.Y.); xjwqy922@163.com (Q.W.); 2 Institute of Occupational Disease Prevention, Jiangsu Provincial Center for Disease Control and Prevention, Nanjing 210009, China; E-Mails: blzhunjmu@163.com (B.Z.); hd-zhang@263.net (H.Z.); dingbangmei@163.com (B.D.)

**Keywords:** prevalence characteristics, coal workers’ pneumoconiosis, China

## Abstract

Coal Workers’ Pneumoconiosis (CWP) is the primary occupational disease in China. However, information about the definite prevalence of CWP is only partially available. The aims of our study were to assess the prevalence characteristics of CWP in a state-owned coal mine, evaluate the effects of control measures and develop further preventive strategies for CWP. The total study population included 495 cases who were diagnosed with CWP from the construction of this coal mine to the end of October 2014. Individuals’ information, including duration of dust exposure, job titles, age as first diagnosis, stages of CWP, CWP progress, complications with pulmonary tuberculosis, death and others were collected and analyzed. The results showed that 71.11% of 495 CWP cases were stage I and 90.71% were involved in tunneling or coal mining. The mean dust exposure period in CWP patients was 26.7 years, the mean latent period was 29.3 years and the mean diagnosed age was 50.3 years old. The proportion of CWP diagnosed after ending dust exposure were remarkably increased with the time passing. Among the CWP cases, 36 (7.27%) were complicated with pulmonary tuberculosis. The mortality of patients with stage III was the highest (60.71%) (*p* < 0.0001). Our data obviously show that more strict policies to protect coal miners are needed to be implemented in China, especially for tunneling and mining workers.

## 1. Introduction

Coal workers’ pneumoconiosis (CWP) is the most serious occupational disease occurring in underground coal miners. The leading cause of CWP is prolonged exposure to airborne coal mining dust which contains high concentrations of free crystalline silica [1]. CWP is an irreversible disease, characterized by inflammation and development of progressive pulmonary fibrosis, which can eventually lead to respiratory failure and no effective treatment for silicosis has been identified to date [2,3].

China is one of the world countries with the most cases of pneumoconiosis, dust exposed population and new cases annually [4,5], which accounted for 87.72% of all reported occupational diseases in 2013. In the developed countries, the incidence of CWP for coal miners has been low because of the use of effective dust control measurements [6]. However, in the developing countries, the incidence of CWP is still at high levels, especially in China [7]. Data from the official website of the China National Institute of Occupational Health and Poison Control showed that 23,152 new cases were diagnosed with pneumoconiosis in 2013, of which 13,955 (60.28%) had CWP and 8,095 (34.96%) had silicosis, that is, CWP accounted for approximately 50% of the total new cases of pneumoconiosis in China. The new occupational cases were mainly from the coal industry (57.13%) [8]. In order to take efficient measures to prevent and control the occurence of CWP, we should explore the prevalence characteristics of CWP, for instance, duration of dust exposure, stages of CWP, job titles, duration of the interphase of CWP progress, complications, *etc.*

The mines were divided into state-owned mines, joint-stock mines and private mines according to the economic types. Generally speaking, the conditions and safety measurements in state-owned mines are better than those of private mines. Here, we investigated the prevalence of pneumoconiosis in a state-owned mine which was located in Jiangsu Province in the east of China. The accumulated number of workers in the underground coal mine with dust exposure was 8928, including employees on the job and retired. The overall prevalence of CWP was 5.54%. To some extent, this example could represent all of the large state-owned coal mines in the east of China. This coal mine is equipped with advanced mining equipment and effective measures to control dust exposure. The prevalence characteristics of CWP could provide some clues to control both the dust exposure levels and the incidence of CWP.

## 2. Materials and Methods

### 2.1. Study Population

Since the coal mine was started up, 495 miners were diagnosed as CWP cases during 1963 to 2014. All of them were recruited in the study. In brief, the full-sized chest X-ray with good quality was performed for reconfirming the diagnoses of CWP based on the China National Diagnostic Criteria for Pneumoconiosis [9], which are the same as the 1980 International Labor Organization (ILO) Classification of pneumoconiosis in the judgment of opacity profusion [10]. The diagnoses were made independently by three certified doctors on the basic of occupational history, physical examination, chest radiograph and pulmonary function tests. The cases were classified into stage I, stage II and stage III according to the size, profusion and distribution of opacities on the chest radiographs.

### 2.2. Data Collection

Subjects’ information included age, job titles, dust exposure period, age at first silica dust exposure, age at first diagnosis, CWP stage, CWP progress, pulmonary tuberculosis complications, death, *etc.* the smoking status data was not complete. There were a lot of job classifications in the underground mine, so the miners were firstly sorted into two groups according to the exposure dust levels and concentrations of silica in this study: one type was tunneling and mining miners, the other type was transport and helping miners. Tunneling and mining miners’ job included primarily creating tunnels, drilling, blasting and cutting of the coal. The transport and helping functions referred to the areas of transportation, maintenance, and electromechanical equipment operation, where dust was not produced directly.

### 2.3. Statistical Analyses

Age at first diagnosis was defined as the age when the worker’s chest radiographs had been diagnosed as stage I or higher for the first time. The duration of dust exposure was calculated by the history from the starting date to the date of ending dust exposure work, the latent period was calculated by taking the time from the date of first exposure to dust to the date of CWP diagnosis. All statistical procedures were performed using SPSS 16.0 (SPSS Institute, Inc., Chicago, IL, USA). Data were analyzed by two-sample t-test, ANOVA or Kruskal-Wallis rank test for continuous data, and χ^2^-test for the categorical variables. All statistical tests were two-sided at a significance level of 0.05.

## 3. Results

Since the coal mine was constructed, a total of 495 CWP cases were diagnosed and divided into three categories, stage I, stage II and stage III, according to the Chinese diagnostic criteria. Among them, 90.71% were involved in tunneling or mining for at least one year. At the end of Oct, 2014, 71.11% of total cases were at stage I and the remainder were at stage II (17.58%) and stage III (11.31%). The mean ages at the first diagnosis were significantly different among three categories, in which the stage I, stage II and stage III were 52.3, 46.8 and 43.3 years old, respectively (*p* < 0.0001). Both the means of exposure duration and latent period had the same trends that stage II and III were significantly shorter than that of stage I. The youngest case was 27 years old and the shortest exposure duration and latent period were 6 years and 7 years, respectively (Table 1).

**Figure 1:**
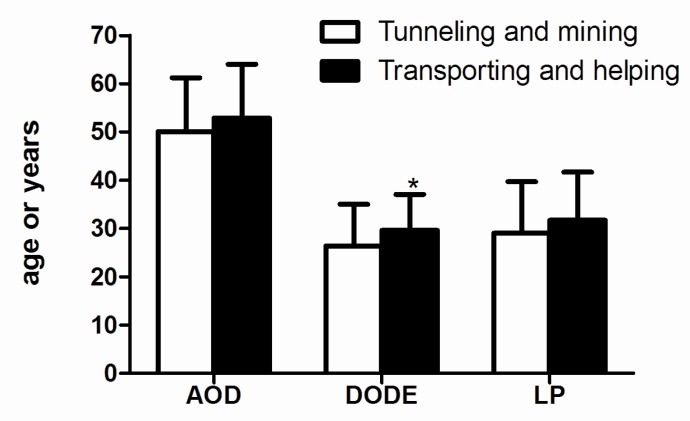
The age of diagnosis, duration of dust exposure and latent period of CWP patients in different job titles. Abbreviations: AOD, age of diagnosis; DODE, duration of dust exposure (years); LP, latent period (years). * *p* < 0.05 compared with tunneling and mining workers.

As shown in Figure 1, there were no significant differences between the tunneling and mining workers and the transporting and helping workers at the mean ages and latent period, but the exposure duration of the former ones (26.4 years) was shorter than that of later ones (29.6 years). 

The mean age at diagnosis for tunneling and mining workers was 50.1, and for transporting and helping workers it was 52.9. The mean duration of dust exposure for tunneling and mining workers was 26.4 years, and for transporting and helping workers it was 29.6 years. The average latent period of tunneling and mining workers was 29.0 years, and for transporting and helping workers it was 31.7 years.

The mean and median of CWP age, exposure duration, and latent period for the different periods are shown in Table 2. The mean age at diagnosis increased significantly over time with the mean age of diagnosis being 41.4 years old before the 1980s, 45.8 years old in the 1980s, 50.8 years old in the 1990s and 60.0 years old after 2000 (*p* < 0.0001). Meanwhile, the mean exposure duration also significantly increased over time with the means in the 1980s, 1990s and after 2000 being 25.2, 26.6 and 32.0 years, respectively, compared to 21.8 years before the 1980s and furthermore the mean after 2000 was found to be much longer than that of any other era. The mean latent period before the 1980s was 21.5 years, 25.2 years in the 1980s, 29.8 years in the 1990s and 37.6 years after 2000, respectively, and there was a marked increase over the past years (*p* < 0.0001).

**Table 1:** The distributions of age of diagnosis, duration of dust exposure, latent period and job titles in different stages of CWP.

CWP Stage	N	Constituent Ratio (%)	Age of Diagnosis			Duration of Dust Exposure (yrs)			Latent Period (yrs)			Job Titles	
			Mean *±* SD	Median	Range	Mean *±* SD	Median	Range	Mean *±* SD	Median	Range	Tunneling and Mining (%)	Transporting and Helping (%)
I	352	71.11	52.3 ± 11.0	52.0	27.0–81.0	27.7 ± 8.4	31.3	6.0–51.4	31.0 ± 10.7	30.0	7.0–60.0	313 (88.92)	39 (11.08)
II	87	17.58	46.8 ± 10.4 ^a^	51.0	29.0–74.0	25.1 ± 8.1 ^a^	23.9	8.4–55.0	26.8 ± 10.0 ^a^	24.0	9.0–55.0	82 (94.25)	5 (5.75)
III	56	11.31	43.3 ± 8.6 ^a,b^	42.0	29.0–66.0	22.9 ± 8.7 ^a^	21.3	10.0–46.7	22.8 ± 8.3 ^a^	21.5	10.0–46.0	54 (96.43)	2 (3.57)
Total	595	100.00	50.3 ± 11.1	53.0	27.0–81.0	26.7 ± 8.5	29.0	6.0–55.0	29.3 ± 10.7	31.0	7.0–60.0	449 (90.71)	46 (9.29)
*F or χ^2^*			23.514			9.537			18.344			4.805	
*p*			<0.0001			<0.0001			<0.0001			0.090	

^a^ *p* < 0.05 compared with stage I; ^b^ *p* < 0.05 compared with stage II; yrs: years.

**Table 2:** The distributions of age of diagnosis, duration of dust exposure and latent period of CWP in different eras of diagnosis.

Time Range	N	Age of Diagnosis			Duration of Dust Exposure (yrs)			Latent Period (yrs)		
		Mean *±* SD	Median	Range	Mean *±* SD	Median	Range	Mean *±* SD	Median	Range
<1980	91	41.4 ± 7.16	40.0	29.0–66.0	21.8 ± 7.5	20.1	8.4–46.7	21.5 ± 7.2	20.0	9.0–46.0
1980–	69	45.8 ± 8.2 ^a^	44.0	28.0–71.0	25.2 ± 8.0 ^a^	24.3	8.7–51.4	25.2 ± 7.7 ^a^	24.0	8.0–51.0
1990–	229	50.8 ± 9.5 ^a,b^	51.0	27.0–80.0	26.6 ± 7.8 ^a^	27.0	6.0–45.0	29.8 ± 9.3 ^a,b^	30.0	7.0–60.0
2000–	106	60.0 ± 10.9 ^a,b,c^	59.0	37.0–81.0	32.0 ± 8.5 ^a,b,c^	33.0	6.5–55.0	37.6 ± 11.6 ^a,b,c^	35.5	8.0–57.0
*F*		72.808			28.140			54.316		
*p*		<0.0001			<0.0001			<0.0001		

^a^ *p* < 0.05 compared with years of diagnosis before 1980; ^b^ *p* < 0.05 compared with 1980–; ^c^ *p* < 0.05 compared with 1990–.

There were 196 (39.60%) CWP cases diagnosed after ending dust exposure, and the interphase ranged from 1 year to 37.5 years (mean 9.5 years). The ratios increased remarkably with the time passed (*p* < 0.0001) and notably after 2000, 75.47% cases were diagnosed after ending dust exposure (Table 3).

**Table 3:** The distributions of period after ending exposure of CWP in different diagnosis periods.

Time Range	N	Period after Ending Exposure (yrs)			
		N (%)	Mean *±* SD	Median	Range
<1980	91	8 (8.79)	7.2 ± 4.8	8.3	1.0–13.6
1980–	69	11 (15.94)	6.3 ± 5.5	6.0	1.0–16.0
1990–	229	97 (42.36)	9.8 ± 8.0	8.1	1.0–35.0
2000–	106	80 (75.47)	9.7 ± 7.6	10.0	1.0–37.5
*χ^2^ or F*		102.450	0.949		
*p*		<0.0001	0.418		

CWP progress is shown in Table 4. There were 419 CWP cases who were first diagnosed as stage I and 61 (14.56%) cases progressed from stage I to stage II up to Oct, 2014, in which the interphase ranged between 0.2 and 24 years (median 5 years). Similarly, a total of 38 CWP cases were first diagnosed as stage II and 12 (31.58%) cases progressed from stage II to stage III and the interphase ranged between 0.2 and 2 years (median 7.5 years). Moreover, 26 CWP cases progressed from stage I to stage III, including 17 cases that progressed from stage I to stage II and then to stage III and the interphase ranged between 2 and 32 years (median 17 years). 

**Table 4:** The duration of CWP progress.

CWP Progress	N	Median (yrs)	Range (yrs)
I→II	61	5.0	0.2–24.0
II→III	12	7.5	0.5–21.0
I→III	26	17.0	2.0–32.0
Total	97	7.0	0.2–32.0

As shown in Table 5, 36 (7.27%) CWP cases were complicated with pulmonary tuberculosis. The incidence of tuberculosis in stage I was 1.99%, 11.49% in stage II and 33.93% in stage III, in which the incidence increased significantly with the CWP progress (*p* < 0.0001). No difference was observed among the job titles. Moreover, the incidence gradually declined with the time passing (*p* < 0.0001), and it was lower than 5% in CWP cases after 1990.

**Table 5:** CWP with pulmonary tuberculosis complication.

Group	N	Pulmonary TB		*p*
		N	%	
Stage				<0.0001
I	352	7	1.99	
II	87	10	11.49	
III	56	19	33.93	
Job titles				0.392
Tunneling and mining	449	34	7.57	
Transporting and helping	46	2	4.35	
Eras of diagnosis				<0.0001
<1980	91	15	16.48	
1980–	69	12	17.39	
1990–	229	8	3.49	
2000–	106	1	0.94	

Ninety five (19.19%) CWP cases were dead, including 35 (9.94%) cases with stage I, 26 (29.89%) case with stage II and 34 (60.71%) cases with stage III (Table 6). The mortality increased with CWP progress (*p* < 0.0001) and decreased with the time passed (*p* < 0.0001). In addition, the mean death age of CWP was 56.8 years, which was much shorter than the mean age of CWP survivors (71.6 years).

**Table 6:** The death status of CWP cases.

Group	N	Death		*χ^2^*	*p*
		N	%		
Stage				88.085	<0.0001
I	352	35	9.94		
II	87	26	29.89		
III	56	34	60.71		
Job titles				0.005	0.946
Tunneling and mining	449	86	19.15		
Transporting and helping	46	9	19.57		
Eras of diagnosis				138.683	<0.0001
<1980	91	55	60.44		
1980–	69	17	24.64		
1990–	229	23	10.04		
2000–	106	0	0		

## 4. Discussion

CWP is a chronic occupational pulmonary disease without effective treatment. The long-term inhalation of dust exposure can activate the inflammation response of the alveoli, lead to irreversible pulmonary damage, and ultimately CWP [3,11,12]. In our study, most of the diagnosed patients were CWP patients in stage I. As CWP progressed, the onset age was younger and younger. Patients with stage II/III had a shorter duration of dust exposure and latent period than those with stage I CWP. The age at diagnosis, duration of dust exposure and CWP latency period could reflect the risk of dust exposure. The workers with the youngest age at diagnosis, the shortest duration of dust exposure and latent period, had the highest risk of dust exposure. Coal miners with distinct durations of dust exposure and occupational categories may be at diverse levels of risk for CWP [13]. This may due to the effective approaches for dust control and adequate prevention measures in the coal mine.

Our data showed that the majority of CWP patients were tunneling and coal mining workers. In general, tunneling and mining miners had highest levels of dust exposure and the highest risk for CWP [13,14]. The highest numbers of patients with the higher stages corresponded to the tunneling and mining miners. The rate of tunneling and mining miners in patients with stage III was 96.43%. The mean duration of dust exposure for CWP patients among tunneling and mining miners was shorter than that in transport and helping miners, showing that the tunneling and mining miners should be protected from CWP in the coal mine. The above results were in agreement with previous studies, which demonstrated that work types and duration of dust exposure are vital factors for the occurrence of CWP [2,15,16], therefore, strengthening management, implementation of effective wet work, adequate individual protective equipment and automation and the closed production of dust are the main measures for decreasing the risk of CWP in the tunneling and mining workplaces [17]. Monitoring and dust control need to be improved in the tunneling and mining areas [18], and a safe workplace and environment should be ensured [19].

With the delayed age of diagnosis, the mean age for CWP diagnosis and the latent period increased. The mean duration of dust exposure for CWP patients diagnosed after 2000 was longer than that before 2000, showing that effective dust suppression measures, the use of qualified and valid personal protective equipment help delay the onset of CWP.

In the present study, 196 (39.6%) CWP cases were diagnosed after ending dust exposure. The interphase ranged from 1 year to 37.5 years (mean 9.5 years). Miners could develop CWP 15 to 20 years after being separated from dust exposure, especially at low concentrations, identified as late-onset pneumoconiosis [18]. The miners from environments with the highest concentration and content of free silica had the greatest probability for the development of respiratory diseases, such as CWP [13,20]. Given that the damage to the organism caused by dust inhalation is a long-term process, the risk for pulmonary fibrosis could remain and the progression could continue even after dust exposure removal [21,22]. Consequently, the physical examination for terminating dust-exposed workers should be performed regularly, and the frequency of the examination in the first 10 years out of dust exposure should be increased. This will help facilitate early detection and timely treatment of CWP, improve the quality of life for CWP patients and prolong their lifespan. 

The interphase of CWP progress could reflect the speed of the CWP deterioration [23]. This is mainly related to the cumulative dust inhalation, the content of free silica, the severity of the first diagnosis, pulmonary tuberculosis and the duration after dust exposure removal [24]. Our study indicated that the mean duration for the interphase from stage I to stage II was 5.0 years, the duration from stage II to stage III was 7.5 years and stage I to stage III was 17.0 years. In consistency with the data of Wang *et al*. and Guan *et al*. [23,25], the duration for the interphase of CWP progression was not associated with years to diagnosis. The onset age did not increase with a shorter of duration of the interphase of CWP progress. This may be due to the fact that there were only 61 cases who progressed from stage I to III. The sample size was so small that the duration for the progress of CWP had a non-normal distribution and it might not adequately reflect the characteristics of the CWP interphase. 

CWP patients often present complications with some other diseases, including tuberculosis, cancer or autoimmune diseases [20,26]. Tuberculosis was the most common complication [27]. The rate of CWP cases complicated with pulmonary tuberculosis was 7.27% in the coal mine, which was reduced by 8.55% compared with the proportion of 15.82% in 1986. The rates of cases with CWP stage I, II and III complicated with pulmonary tuberculosis were1.99%, 11.49% and 33.93%, respectively, which was lower than the 14.82%, 14.74% and 34.60% numbers in 1986 [18]. The patients with higher stage had an increased risk of tuberculosis. Silicosis was reported to coexist with tuberculosis. The upper airway stimulated by dust for a long time would lose the defense ability and become susceptible to be invaded by *Mycobacterium tuberculosis*. Firstly, the fibrosis in the lung of silicosis patients hindered the circulation in blood and lymph, reducing the defense capability against *M. tuberculosis*. Secondly, the free silica in dust plays a toxic role on macrophages, weakening the capability of phagocytosis and the *Mycobacterium tuberculosis* killing ability of macrophages. Finally, the space where coal miners work is relatively small, increasing their opportunity and time of contact with *Mycobacterium tuberculosis*, to which the chance of *Mycobacterium tuberculosis* spreading in the air must be added. Some data indicates that pneumoconiosis complicated with tuberculosis was not a simple combination of pneumoconiosis and pulmonary tuberculosis. It was an integral part of the pathological process of pulmonary fibrosis and lung parenchyma destruction, with mutual promotion and influence [28,29]. Hence, tuberculosis prevention education is needed in the workers exposed to dust and pneumoconiosis patients. Meanwhile, cardiopulmonary exercises are demanded for decreasing the chance of tuberculosis infection. Tuberculosis screening frequency should be increased for patients with pneumoconiosis in Stage II and Stage III. Active treatments and better management are required for pneumoconiosis patients suffering from tuberculosis to reduce the mortality. Tuberculosis patients should leave jobs with dust exposure, decreasing any further danger of exposure to dust to the patients’ life.

CWP patients with the highest stage had the highest mortality. This phenomenon may result from the fact that CWP patients are susceptible to pulmonary tuberculosis, respiratory failure and chronic pulmonary heart disease [30]. The mean age of surviving CWP patients was 71.6 years old, which was similar to the age of the normal population, demonstrating that the control of coal dust pollution, personal protective measures, CWP screening and better levels of health care had been developed at the enterprise.

Several limitations of our study should be addressed. Ninety five CWP patients had died and the majority of surviving CWP patients had retired, scattered, and were contacted by a single way. Therefore, it was difficult to investigate their smoking status one by one, leading to incomplete smoking status information, so the impact of smoking on the incidence of CWP in the coal mine was not analyzed. We plan to collect relevant information in future CWP physical re-examination and cohort studies. Smoking was significantly associated with pneumoconiosis. Smokers with long-term cigarette use were reported to be more susceptible than non-smokers to the associated silicosis [31]. Hessel *et al*. [32] showed that hydrocyanic acid in the tobacco smoke could harm the bronchial epithelium and cilium, reducing the capability to remove dust. Some population-based studies have confirmed that smoking promoted the incidence of silicosis [31]. Consequently, smoking cessation would have a positive effect in the prevention of CWP and it should be encouraged in coal miners. In addition, considering that mine dust concentrations were not monitored before 1974, the cumulative dust exposure could not be calculated for all patients with CWP. 

## 5. Conclusions

The present study assessed the prevalence characteristics of CWP in a state-owned coal mine in China. The findings showed that the majority of CWP cases were in stage I and tunneling or coal mining workers. Moreover, the CWP cases in stage III were more susceptible to complicate with pulmonary tuberculosis and had the highest mortality, compared with other CWP cases. Efforts should be made to improve dust control measures, especially in the tunneling and coal mining workplace. Additionally, further tuberculosis in CWP cases should be prevented and treated carefully, particularly for severe CWP cases. 
